# Innovation without integration

**DOI:** 10.1038/s41746-020-0220-z

**Published:** 2020-02-03

**Authors:** Adam B. Cohen, Seth S. Martin

**Affiliations:** 1 The Johns Hopkins University Applied Physics Lab (APL), Health Technologies, National Health Mission Area, 11100 Johns Hopkins Rd, Laurel, MD 20723 USA; 2 Department of Neurology, The Johns Hopkins Hospital, 1800 Orleans Street, Baltimore, MD 21287 USA; 3 Division of Cardiology, Department of Medicine, The Johns Hopkins Hospital, 600 N. Wolfe Street, Baltimore, MD 21287 USA

**Keywords:** Health care, Therapeutics

The successful introduction of Edison’s incandescent light bulb did not rely on available infrastructure, which was designed around gaslight and candlelight. It required a new foundation—electrical power-generating stations, electricity distribution and delivery systems, overhead wires connecting stations and homes, and massive funding from J.P. Morgan and the Vanderbilts.^1^

Solving problems with new approaches comes in two extreme forms. The *assimilation model* builds on the existing foundation. It assumes the core pieces of the solution framework remain in place. Typical telemedicine services leverage, adapt to, or depend upon extant aspects of the healthcare system like clinics, hospitals, electronic medical records, and clinicians. Alternatively, the *errant model* makes no such assumption. It looks beyond the existing foundation and recognizes that the optimal solution may need a new foundation. Many existing direct-to-consumer prevention and wellness innovations require few to no components from the standard system. These include virtual classes with certified instructors, social motivation digital platforms, and app-derived bespoke recommendations driven by patient-generated wearables data. If successful, such products could obviate the future need for medical diagnostics and management. The errant model not only improves the field, it redefines it.

In this issue of *npj Digital Medicine*, Gordon et al.^2^ propose a thoughtful framework for mobile health apps to better enable integration into the existing clinician-driven healthcare system. The framework explores education and awareness, digital app formularies, workflow and electronic medical record integration, payment models, and patient/provider support. Their recommendations will be invaluable for apps and digital health technologies adhering to the assimilation model.

Their approach provides pragmatic steps to get health apps into clinical practice. For clinician education, the authors suggest formal training in digital health as part of ongoing professional education and certifications to prescribe specific apps. Such app prescriptions may be guided by a digital formulary that offers a short list of apps vetted for safety, efficacy, interoperability, and price. App prescriptions themselves could simulate traditional medication prescriptions, e-prescribed in the electronic medical record, with directions for use and an approved clinical indication.

Their suggestions cater nicely to clinicians. After app prescription, app usage data and patient-generated health data outputs could flow back to the electronic medical record. To make apps useful for patients in this framework, electronic medical record integration is a key component.

The errant model, however, requires less, little, or no such integration. This model may create a better user (provider *and* patient) experience and faster uptake. The spectrum of useful apps will span these models^3^ and thus, employ varying degrees of integration. Here, we explore varying levels of digital health technology integration with the electronic medical record as well as other components of the existing healthcare system, including clinicians themselves. Such components include the major cost drivers in the current system, such as the services (clinicians, administration); structures (clinics, wards); and technologies (electronic health record, facility-associated diagnostic machines) that compose it.^4^

We believe prevention-focused digital health technologies are currently most able to avoid integration with the existing healthcare system. This is in line with digital health trends we observe, whereby prevention products directly appeal to consumers outside their normal experience within the healthcare system. It also fits our understanding of what the current healthcare system mainly provides—detection or diagnosis and management over prevention services.^5,6^ Conversely, to be successful, management-focused digital health technologies presently require higher levels of integration into the current system. In the middle, detection-focused digital health technologies may only require integration after detection of possible disease occurs. Then, the typical components of the system, like clinicians and our facilities, take over, to confirm disease and provide management. Thus, a varying ease of low integration exists across this healthcare continuum (Fig. 1).

**Fig. 1:**
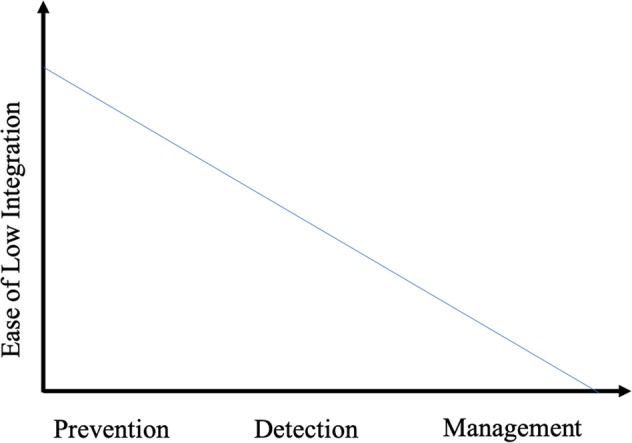
The ease of low integration across the healthcare continuum.

## High integration

High-level integration may take years to achieve. Assimilating into the healthcare system can be slow—very, very slow. Apps that ingest data from or provide data to the electronic medical record require this level of integration. This typically requires consideration and approval from myriad stakeholders ranging from local hospital committees to national regulatory bodies like the Food & Drug Administration (depending on the technology’s intended purpose).

Consider a clinician-prescribed blood pressure tracking app, to be used by the patient, which allows high-frequency home recordings. The clinician can track, be alerted to, or act on the results with a high level of integration into many electronic medical record features, including vitals flow sheets, billing, and patient–provider communication. This also requires high integration into the clinician’s workflow such as schedule and data review. Without explicit processes to monitor and act on these data, inaction ensues.

A medical coding query platform smartphone app, as another example, may prompt a clinician to clarify a diagnosis. This app, which ingests electronic medical record data, would typically mandate prolonged review from local hospital leadership, clinician groups, and billing administration in order to integrate. Here, too, the process depends on clinicians. Developers and implementers of such an app must consider clinician schedules, time management, and culture.

## Moderate integration

Instead of fully relying on the entire electronic medical record machinery, such as clinical data visualization dashboards, apps may provide separate viewing interfaces outside the electronic medical record. Such overlays decrease integration requirements and can dramatically improve the user experience—given the notoriously clunky experience offered by most electronic medical records.

Consider again the blood pressure monitoring app. Instead of navigating to blood pressure recordings within the electronic medical record vital signs worksheet, the app offers a visualization and analytics overlay, bypassing the electronic medical record. It anticipates the clinician’s needs, displaying only useful data such as graphical forms of blood pressure trends, clinically important peaks and troughs, and associations with medication compliance. Given clinical documentation requirements, a moderate level of digital integration would satisfy communication and billing needs, such as depositing a data summary back into the clinical record.

Some digital clinical risk calculators, such as the American College of Cardiology/American Heart Association Pooled Cohort Equations, represent another moderate integration example. They gather clinical data from and generate risk estimate documentation into the digital health record. Like the visual overlay example, the risk calculator interface, too, can exist outside the electronic medical record.

Watches with embedded optical sensors that track cardiac rhythms are sold directly to consumers. This model only aims for interaction within the healthcare system after detection of atrial fibrillation.^7^ Following detection, the patient’s care provider or providers would assume responsibility to partner with the patient on diagnostic confirmation and management.

## Low integration

Apps operating outside clinician workflow, including many patient-facing apps, may require low or no integration. Attempts to integrate them might interfere with or outright quash use, delight in the experience, and performance. A digital joint clinic, for example, may provide patient guidance to manage osteoarthritis. It aims to prevent complications requiring care escalation such as joint replacements. Here, guidance in the form of remote physical therapy, weight control, and basic medication advice requires no input from the clinicians within the patient’s healthcare system (but may have input from clinicians who are part of the company), nor transfer of data into the electronic medical record.

Direct-to-consumer apps, in which patients are both purchaser and end-user, are the most currently relevant group in this low integration category. Fitness-promoting apps, all of which have a disease prevention promise, require little clinician buy-in, integration with clinicians’ workflow, or insurance approval. Although actual health benefits may not exist or have yet to be proven,^8^ this approach currently requires no integration with the electronic medical record or the clinician. As technologies become increasingly autonomous for disease prevention, detection, and management, integration should wane. A virtual diabetes human coach who provides guidance based on wearable glucose patient sensors, for example, could be replaced by an artificial intelligence-powered machine coach.

## Conclusion

The wave of apps and other digital health technologies may take one of two distinct paths. Both may help patients. The first path is integration into the existing infrastructure, including the electronic medical record. Clinicians and administrators may need to modify workflows to incorporate these new offerings, including an apps formulary.

This can create friction, however. Developing system requirements for digital health technologies^9^ may reduce friction by fostering technology assimilation and usefulness under the existing paradigm. Many digital health technologies take this path. They range from physician-prescribed therapeutic apps to a host of other technology offerings such as medical ride hailing apps and automated scribes. All essentially maintain the status quo, but do it in a new way and chip away at efficiency, access, scale, and efficacy.

The second path ignores, or largely ignores, what exists. In fact, what exists today might not be effective or efficient: Patients have difficulty accessing clinical services.^10^ Care and cost quality varies by hospital, region, and provider.^11,12^ Costs climb uncontrollably.^13^ Doctors hate their electronic medical records.^14^ Changing this reality may require severe disruption making the existing model obsolete.^15^ Of course, disruption need not require digital technologies, which could be barbers, not physicians, promoting better health and hypertension control.^16^

We acknowledge these paths, assimilation and errant, cross. New technologies and methods will fall between the two models or incorporate both. Yet, if Edison’s bulb represents the errant model, we believe the medical community will tend toward adopting or developing brighter burning candles and not the electric bulb. As care moves outside the hospital and clinic, and new technologies empower patients and their families, a view through the errant model lens is in order.

As with Edison’s bulb, successful wide-scale implementation of the errant model may demand new infrastructure, deep investment, and great disturbances in the status quo. We cannot foresee all possibilities, but imagine impactful digital health innovations will step outside the existing framework, appeal directly to patients, and remove clinicians and their systems from the loop. Prevention-focused technologies may be the most likely forerunners, followed by detection- and then management-focused technologies.

Indeed, Gordon and colleagues call attention to insufficient aspects of the existing foundation. The healthcare system is not adequately configured to provide technical support to patients using digital health apps, for example. This has led to the emergence of medical “genius bars” to support installation and support for digital health interventions in select hospitals.^17^ Perhaps more than fitting patients into the existing foundation, these are early signals of the disruption ahead.

Innovators *within* the healthcare system naturally find ways to improve existing delivery methods—or determine how innovations fit into existing processes. It is less natural to find replacement methods, some of which would replace our own existence. Integration may be practical, but the biggest, most useful innovations, like Edison’s light bulb, find another approach. Our way into the digital age requires more than candlelight.
